# Microwave-Assisted Synchronous Nanogold Synthesis Reinforced by Kenaf Seed and Decoding Their Biocompatibility and Anticancer Activity

**DOI:** 10.3390/ph15020111

**Published:** 2022-01-18

**Authors:** Md. Adnan, Ki-Kwang Oh, Azamal Husen, Myeong-Hyeon Wang, Madhusudhan Alle, Dong-Ha Cho

**Affiliations:** 1 Department of Bio-Health Convergence, College of Biomedical Sciences, Kangwon National University, Chuncheon 24341, Korea; mdadnan@kangwon.ac.kr (M.A.); nivirna07@kangwon.ac.kr (K.-K.O.); 2 School of Public Health, Wolaita Sodo University, Wolaita Sodo 138, Ethiopia; adroot92@yahoo.co.in; 3 Department of Biomedical Science & Institute of Bioscience and Biotechnology, Kangwon National University, Chuncheon 24341, Korea; allemadhusudhan@gmail.com

**Keywords:** kenaf seed, gold nanoparticles, biocompatibility and anticancer activity

## Abstract

The combination of green-nanotechnology and biology may contribute to anticancer therapy. In this regard, using gold nanoparticles (GNPs) as therapeutic molecules can be a promising strategy. Herein, we proposed a novel biocompatible nanogold constructed by simply microwave-heating (MWI) Au^3+^ ions and kenaf seed (KS) extract within a minute. The phytoconstituents of KS extract have been utilized for safe synthesis of gold nanoparticles (KS@GNPs). The biogenic KS@GNPs were characterized by UV-vis Spectra, TEM, HR-TEM, XRD, FTIR, DLS, EDX, and SEAD techniques. The legitimacy and toxicity concern of KS@GNPs were tested against RAW 264.7 and NIH3T3 cell lines. The anticancer efficacy was verified using LN-229 cells. The pathways of KS@GNPs synthesis were optimized by varying the KS concentration (λmax 528 nm), gold salt amount (λmax 524 nm), and MWI times (λmax 522 nm). TEM displayed spherical shape and narrow size distribution (5–19.5 nm) of KS@GNPs, whereas DLS recorded Z-average size of 121.7 d·nm with a zeta potential of −33.7 mV. XRD and SAED ring patterns confirmed the high crystallinity and crystalline face centered cubic structure of gold. FTIR explored OH functional group involved in Au^3+^ ions reduction followed by GNPs stabilization. KS@GNPs exposure to RAW 264.7 and NIH3T3 cell lines did not induce toxicity while dose-dependent overt cell toxicity and reduced cell viability (26.6%) was observed in LN-229 cells. Moreover, the IC_50_ (18.79 µg/mL) treatment to cancer cell triggered cellular damages, excessive ROS generation, and apoptosis. Overall, this research exploits a sustainable method of KS@GNPs synthesis and their anticancer therapy.

## 1. Introduction

The scientific community around the world has been profoundly involved in discovering new facets of nanotechnology through rapid methodological advancements in the area of science and technology [1]. Nanotechnology can be denoted as an art of science that maintains matter in a way that intercepts within the nanoscale range (i.e., 1–100 nm) of one dimension [2]. Particles at the molecular level have varied trailblazing physicochemical properties. Thus, nanoparticles have achieved tremendous growth and procreated novel fundamental and applied frontiers in the biomedical, drug delivery, optics, chemical industries, material science and engineering, medicine, cosmetics, and food industry [3]. The metallic (Ag, Au, and Se etc.) nanoparticles with tunable physical and chemical properties are relatively considered one of the most sophisticated systems for all the functions mentioned above [4].

At present, cancer has been one of the most significant causes of human death around the globe [5]. Over the past two decades, numerous developments and progressions in various cancer treatment approaches (e.g., surgery, radiation, and chemotherapy) have been recognized in hospitals [6]. A great deal has been undertaken to improve these conventional patterns, which already have a range of limitations, including poor efficiency, extreme adverse reactions, and a greater cancer recurrence [7]. Therefore, interventions with fewer adverse effects and better clinical performance need to be developed. Nanomedicine’s present advancement has made it possible for us to produce many innovative nanomaterials for parallel assessment and treatment.

Current nanotech research uses a vast range of emerging nanomaterials in cancer therapy [8]. Gold nanoparticles (GNPs) are mainly used for various diseases, with multiple benefits, as nanomedicine [9]. They can safely be used in systemic circulation due to their stability and size variance, which is why researchers have concentrated on GNPs for future cancer therapy applications [10]. GNPs can be generally produced with a number of shapes (sphere, rod, branched structure, cage-like, etc.), and they can range in size from 1 nm to 100 nm or more. Because of the surface charge, GNPs are comfortably functioned by different types of biomolecules (i.e., drugs, genes, and targeting ligands) [11]. Hence, GNPs are the most responsive material for different biomedical applications considering all these unique features.

Various physical and chemical techniques are implemented for the generation of metal nanoparticles. However, concern remains on the exploitation of toxic chemicals (hydrazine hydrate, ethylene glycol and sodium borohydride) as reducing agents which are found on the surface of the nanoparticles and restricted their biomedical and clinical application due to toxicity issue [12]. In this regard, the introduction of green synthesis in nanotechnology has brought a revolutionary change in the area from non-specific drug delivery to targeted drug delivery [13]. The utilization of natural products as processing materials is a significant part of green nanotechnology that aims to design sustainable nanomaterials to diagnose and treat various diseases. The use of various plant parts (seed, leaves, root, and bark) and their extract as non-toxic and renewable reducing and stabilizing agents for the nanoparticle’s synthesis have been a fascinating idea [13]. The mixing of plant extract solution with metal salt solution and using various synthesis medium can produce the nanoparticles [14,15,16]. Compared to numerous traditional synthesis methods, microwave irradiation (MWI) is an ultrafast and novel synthesis method, induces a high-frequency wave and temperature to the starting materials within a short time [17]. MWI possesses several advantages, including cleaner product formation, controlling the reaction time, ensuring uniform size and crystalline products, and avoiding possible byproducts formation [18,19]. MWI enables an efficient internal dielectric heat by the interaction of microwave energy with a dipole moment of the molecules. During this process, the electric field leads to oscillation and friction between the molecules that converting electromagnetic energy into thermal energy, thus maintain uniform nucleation and growth rates. Such a convenient approach produces a higher degree and uniformed size GNPs [18,19,20].

**Figure 1:**
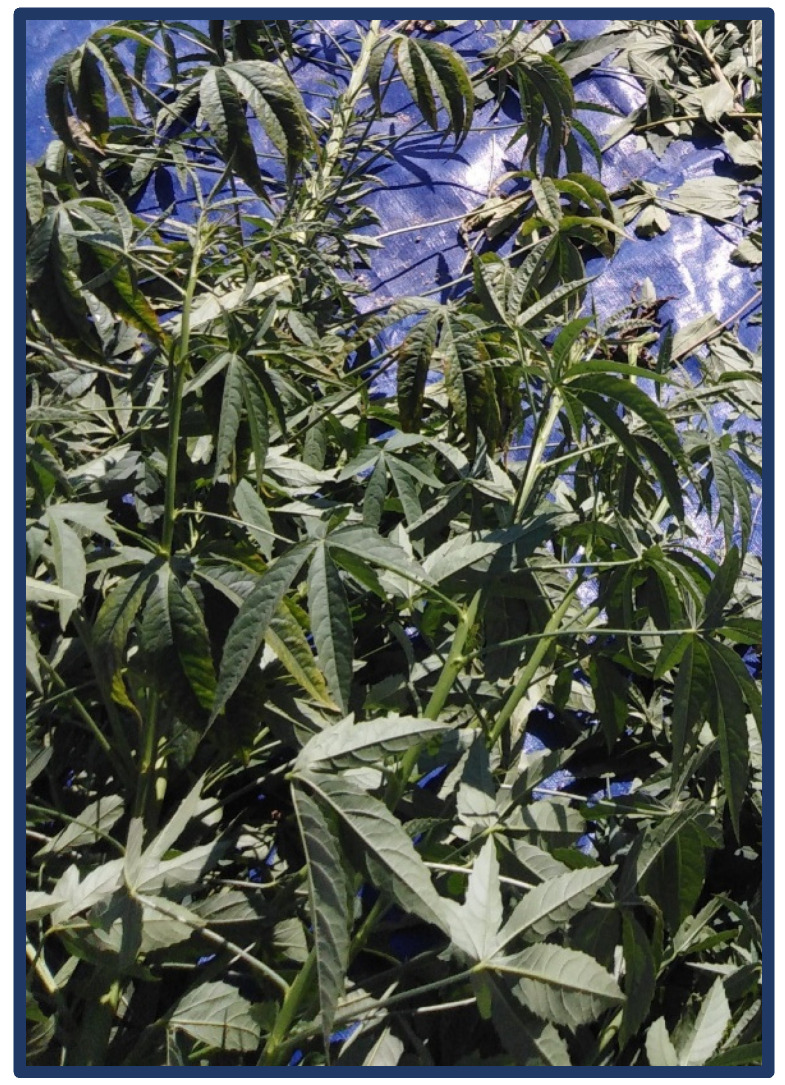
Representation of Kenaf (*Hibiscus cannabinus*).

An industrially valuable plant, “Kenaf (*Hibiscus cannabinus*)” (Figure 1) possesses remarkable anti-cancer, anti-inflammatory, anti-obesity, and antioxidant medicinal properties. Mainly, kenaf seed is the potential source of various health-promoting compounds, such as phenylpropanoid compounds, sterols, kaempferol, and omega-3-fatty acids [21,22,23]. Hence, it has implausible demand in both the food and pharmaceutical industries. Previously, kenaf seed and kenaf seed-based silver nanoparticles exposed promising anti-lung cancer and antibacterial activities [22,24]. Besides, biopolymer mediated nanocomposites were prepared using kenaf seed in order to enhance its antioxidant capacity [21]. However, abundant bioactive compounds and outstanding biological attributes make the kenaf seed a candidate for further research.

In this contribution, we have predominantly focused on the rapid synthesis of kenaf seed capped gold nanoparticles (KS@GNPs) due to their groundbreaking anti-cancer properties. Here, kenaf seed was used as both a reducing and capping agent for the synthesis of GNPs, followed by employing MWI as an easy-to-operate technique. The overall synthesis is simple, cost-effective, faster, and easy to scale up for large scale nanoparticle production. Some essential variables of the nanoparticle synthesis step, such as concentrations of materials and incubation period, were optimized to achieve an ideal outcome. The synthesized GNPs were fully characterized via various analytical techniques. Once fully characterized, their biocompatibility was tested on macrophage (RAW 264.7) cells. Afterwards, the cytotoxicity and anti-cancer activity were evaluated against healthy (Swiss albino mouse embryo tissue, NIH3T3) cells and the human glioma LN-229 cell line.

## 2. Results and Discussion

**Scheme 1:**
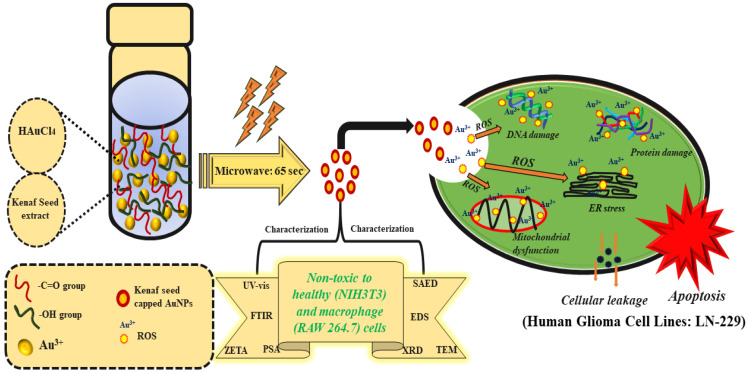
Schematic representation of gold nanoparticles synthesis and their biocompatibility and anti-cancer potentials.

Green synthesis concepts always suggest sustainable and cleaner production of nanoparticles by following state-of-the-art techniques. While avoiding chemical complexity, environmental concern, and toxic way of nanoparticles production, the green concepts have recently been met with wide interest among scientists. Hence, an easy-to-operate system, microwave-assisted synchronous synthesis of kenaf seed reinforced-GNPs was developed and explored their biocompatibility and application in anti-cancer treatment (Scheme 1).

### 2.1. Synthesis Conditions Optimization

**Figure 2:**
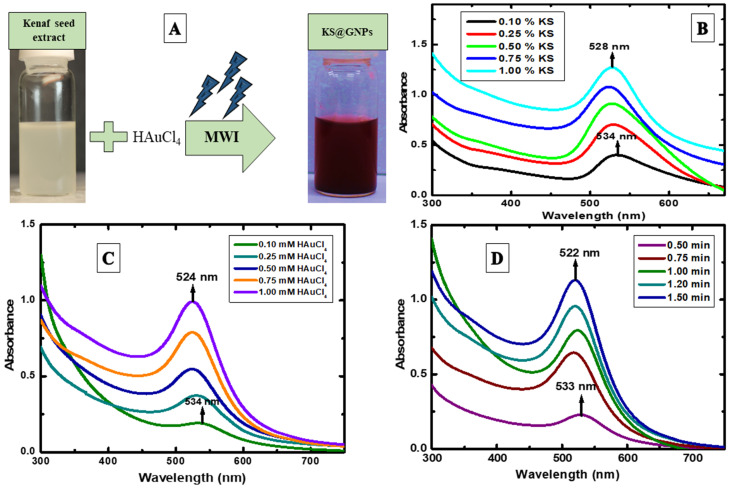
UV-visible spectra of kenaf seed (KS) stabilized GNPs (KS@GNPs). (**A**) Formation of KS@GNPs evidenced after color transformation from light white to brick red. Effect of (**B**) KS concentration (0.1 to 1%), (**C**) HAuCl_4_ concentration (0.1 to 1 mM), and (**D**) microwave irradiation time (0.5 to 1.5 min).

The initial reduction of Au^3+^ by kenaf seed (KS) was confirmed through visual observation of the distinctive dark-purple coloration which arises due to the tiny dimensions of GNPs. At nanometer dimensions, GNPs expose both absorption and scattering effects, for example, an oscillation of the electron cloud at the surface of the nanoparticles resonates and absorbs electromagnetic radiation at a particular energy [25]. This specific phenomenon known as surface plasmon resonance (SPR) which is not only depended on their size and shape, but also on various factors (solvent, temperature, and concentration) which can influence the exact frequency and band intensity. Generally, the dark-purple color appears when the synthesized GNPs possess a spherical shape with a size of less than 60 nm and the SPR peak demonstrates around 500–550 nm [26]. Our synthesized KS@GNPs also manifested such dark-purple color after MWI (0.5 min) treatment (Figure 2A). Such color transformation is usually observed when a metal changes its oxidation state. Presumably, different functional groups present in plant extract caused rapid Au^3+^ reduction as well as contributing to the standard size and shape of GNPs production. 

### 2.2. Effect of Finest KS Concentration 

Further SPR justification of KS@GNPs was monitored by UV-vis spectroscopy which is the most excellent tool to characterize the optical properties and electronic structure of NPs. Figure 2B presented UV-vis spectra of KS@GNPs, which were evidenced after MWI (0.5 min), implemented on the mixture of different concentrations of KS (0.1 to 1%) and constant concentration of HAuCl_4_ (0.5 mM) solution. The detected strong absorption bands due to SPR were mostly appeared in the region of 520 to 540 nm. The SPR intensities were concentration dependent, and peaks were fluctuated with the variation of KS concentrations. At the very low concentration (0.1%), the SPR peak at 534 nm showed less intensity and shifted toward a longer wavelength, indicating increased particle size, poor nucleation, and growth of GNPs; these might be the reason for the inadequate KS concentration for the GNPs formation. However, upon raising with the KS concentrations (0.25 to 1%), the intensity of absorption bands increased while a sharp SPR peak at 528 nm was noted for the maximum concentration (1%) which acted as an effective reductant and stabilizer for controlled sized GNPs formation. Therefore, 1% KS was selected as an optimum concentration for the synthesis of GNPs.

### 2.3. Effect of Finest HAuCl_4_ Concentration 

To finely tune the size of GNPs, we investigated the effects of the different HAuCl_4_ concentrations (0.1 to 1 mM), whereas KS concentration (1%) and MWI (0.5 min) time remained stable. Figure 2C revealed that the absorption peaks and wavelength range significantly differed, and absorbance curve turned out to be more bulging when HAuCl_4_ concentrations were increased. At elevated concentration (1 mM), the SPR peak intensity was sharp while peak position shifted from 528 nm to 524 nm (compared to Figure 2B). According to the Mie’s theory, synthesized NPs demonstrate spherical shape with size-control less than 15 nm when the SPR peak appears between 520 nm to 525 nm. For anisotropic NPs, such as nanorods (NRs), at least two SPR bands are observed depending on the particle’s size and shape [18,27,28]. Based on this theory, it is predicted that our synthesized GNPs possess spherical shape since 1 mM HAuCl_4_ displayed extremely high SPR band at 524 nm; therefore, this optimized HAuCl_4_ concentration is enough for well stabilized and size control GNPs formation.

### 2.4. Effect of Ideal MWI Time

With respect to optimizing the reaction parameters (1% KS and 1 mM HAuCl_4_), another simple strategy to achieve size control of GNPs is to observe the influence of different duration time (MWI times) of synthesis. Here, the initial reduction reaction was evidenced after 0.5 min of MWI time which resulted in color transformation of the solution with the manifestation of a weak and long-wavelength SPR at 533 nm (Figure 2D). However, as MWI time (0.5 to 1.5 min) exposition increased, the reduction reaction (gold ions to GNPs) became more forceful and SPR peaks progressively increased (Figure 2D). An intense SPR peak with shorter wavelength at 522 nm (compare to Figure 2C) was noticed when MWI time was elevated to 1.5 min, ascribing a large amount of Au^3+^ converted into the Au^0^ followed by GNPs formation with suitable particle size. Surprisingly, when KS concentration (>1%), HAuCl_4_ concentration (>1 mM), and MWI time (>1.5 min) were further increased during each optimization period, the peak intensity did not change and wavelength became border. Therefore, the obtained results (optimized reaction parameters and time) established a promising synthesis route for successful GNPs formation which seems to give a finer size control.

### 2.5. Transmission Electron Microscopy (TEM) Analysis of KS@GNPs

**Figure 3:**
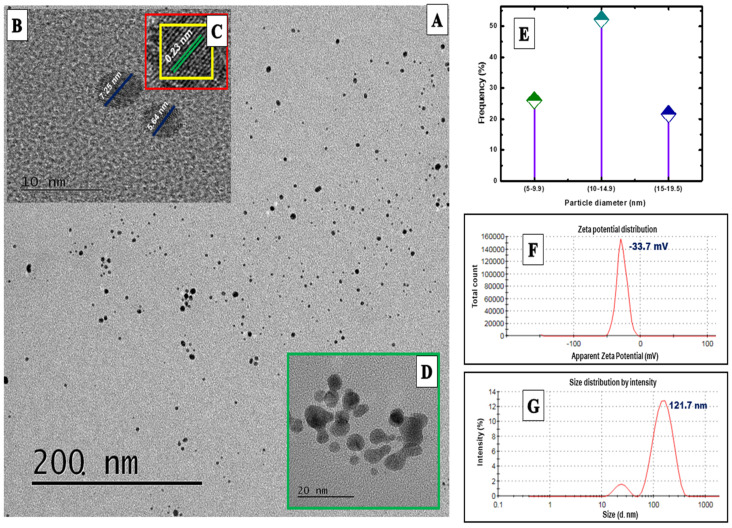
Transmission Electron Microscopy (TEM) image (200 nm) of KS@GNPs (**A**), TEM image at 10 nm (**B**), HR-TEM image (**C**), TEM image at 20 nm (**D**), particle size histogram of KS@GNPs (**E**), Zeta potential value of KS@GNPs (**F**), and particle size distribution of KS@GNPs (**G**).

The morphological features of GNPs synthesized under optimized conditions were investigated by TEM analysis. TEM provides brief information on the size and shape of NPs with very high accuracy on a routine basis. Figure 3A,B,D represented well-separated biosynthesized GNPs having a mono-disperse tendency with a spherical shape that attributed to the stabilized NPs formation, possibly due to the involvement of active functional groups of KS, served as strong reducing and stabilizing agents. The histogram (particle size distribution) constructed by analyzing 90 particles from various TEM images revealed the narrow size distribution range (5 to 20 nm) of KS@GNPs (Figure 3E). Just over a half (52.25%) of NPs was recorded within the 10 to 14.5 nm range, roughly one quarter (26.10%) within the 5 to 9.9 nm range, and almost one quarter (21.65%) within the 15 to 19.9 nm range. The HR-TEM analysis uncovered 0.23 nm space of atomic lattice fringes, which resembled to the lattice distance of plane (111) reflection of FCC crystal structure of Au (Figure 3C). In addition, the effective electric charge on the nanoparticle surface was measured (ζ-potential value) to identify the stability of particles. Nanoparticles are considered to be more stable if two adjacent NPs possess high ζ-potential value of same sign, leading to high electrostatic repulsion between NPs [29]. In our study, the zeta potential value of KS@GNPs was found −33.7 mV (Figure 3F), which indicated enhanced colloidal stability, no agglomeration or flocculation, and well dispersion of GNPs. Besides, the average particle size distribution of KS@GNPs in the solution was determined 121.7 nm (Figure 3G). Importantly, the size of GNPs obtained from DLS was not typical as compared to the size measured with TEM, and it was also noted that DLS always overestimates the NPs sizes [30]. 

**Figure 4:**
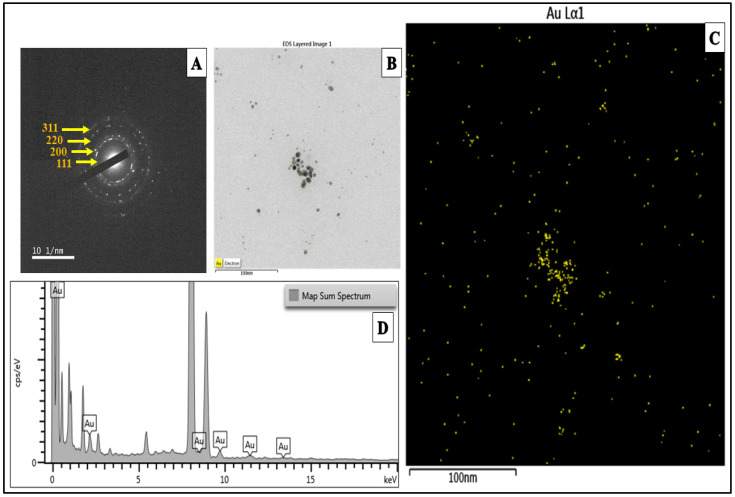
SAED pattern of bio-synthesized KS@GNPs (**A**), Energy Dispersive X-ray (EDX) spectrum of KS@GNPs (**B**,**C**), and EDX based mapping of Au (**D**).

The selected area electron diffraction (SAED) pattern identified the single crystallinity nature of KS@GNPs, detected from the sharp diffraction spots of one particle Figure 4A. The EDX spectrum revealed the elemental composition of GNPs and their surrounding (Figure 4D). The result unambiguously confirmed the peaks of metallic Au atom at 0.5, 2, 8.5, 9.7, 11.5, and 13.5 KeV. The additional peaks were found small to high amounts and attributed to C, O, N, and Cu. The Cu might be originated from copper grid, on which the samples were analyzed [31]. The appeared other elements (C, O, and N) were due to elemental composition of kenaf seed, indicating that the extract effectively involved in the biosynthesis of GNPs. The elemental screening also detected the Au (Figure 4B,C) which was in accordance with the result of TEM analysis.

### 2.6. X-ray Diffraction (XRD) and Fourier Transform Infrared Spectroscopy (FTIR) Analysis

**Figure 5:**
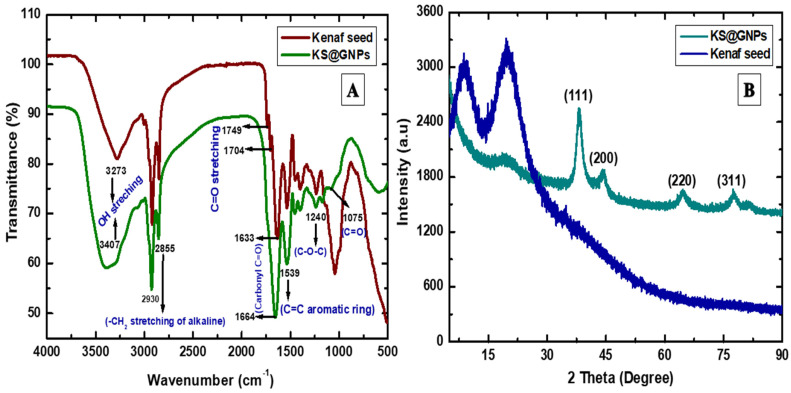
Fourier transform infrared (FTIR) analysis of synthesized KS@GNPs (**A**), and X-ray diffraction (XRD) pattern analysis (**B**).

The crystallographic structure of KS@GNPs was verified by X-ray diffraction (XRD) pattern. As presented in Figure 5B, four characteristics peaks at 2 theta values were observed at 38.35, 44.63, 64.51, and 77.47 which were assigned to 111, 200, 220, and 311 planes, and completely matched the reflections of face centered cubic GNPs when compared these peaks positions with the standard gold metallic form (JCPDS) file No. 04-0784. This similarity indicated that the synthesized KS@GNPs were of pure crystalline in nature. However, the most dominant peak with high intensity and area was observed at 38.35° while other remained poor intense and longer, indicating that the maximum growth of GNPs was 111 plane orientation. 

Here, we obtained FTIR spectra (Figure 5A) that detected the key functional groups responsible for the reduction of Au^3+^ ions and stabilization of GNPs. The FTIR spectrum of KS was complex with various characteristics peaks at the region between 4000 and 500 cm^−1^ wavenumbers. OH stretching (3273 cm^−1^) and -CH2 stretching of alkane (2930 and 2855 cm^−1^) were the major bond vibrations, originating from the lipid compositions of KS. The two major peaks at 1749 and 1704 cm^−1^ were assigned to saturated ester C=O stretching that originated from cholesterol esters or phospholipids part of KS. Also, there was a peak at 1633 cm^−1^ (carbonyl C=O) mainly from protein source of kenaf seed. However, after MWI, the obtained FTIR spectrum revealed some structural modifications as well as interactions between gold ions and functional group of molecules. After synthesis (KS@GNPs), the intensity or area of some FTIR absorption peaks reflected very prominent differences, mainly, a peripheral shift with high area from 3273 to 3407 cm^−1^ and 1633 to 1664 cm^−1^. Besides, peaks at 2930 cm^−1^ and 2855 cm^−1^ were demonstrated high intensity compared to the control (KS). Most importantly, the two individual peaks at 1749 and 1704 cm^−1^ of the KS were disappeared after GNPs synthesis [16,21,24]. These observed band deviations confirmed the co-ordination bond formation with GNPs and OH functional group involved in the gold ion reduction followed by GNPs formation. Here, calculated hydrogen bonding energy from this equation HE = K − 1 (V_o_ − V/V_o_) (Vo is the standard free OH frequency 3650 cm^−1^; V is the hydrogen bonded sample OH frequency; and K is a constant 3.8 × 10^−3^ kJ^−1^) revealed that HE values of KS and KS@GNPs were 27.16 kJ and 17.50 kJ, respectively. Such shifting value of HE pinpointed the hydrogen bond formation between KS@GNPs and OH of KS [18]. Importantly, our previous chromatographic like HPLC analysis of KS revealed a sufficient amount of phenolic acids, including kaempferol, gallic acid, tannic acid, sinapic acid, catechin hydrate, and 4-hydroxy benzoic acid [21], which are an abundant source of OH functional group, might be responsible for the reduction of Au^3+^ ions and stabilization of GNPs. However, we also observed some overlapping peaks of KS and KS@GNPs, which indicated the KS remained intact even after MWI.

### 2.7. Biocompatibility Assessment of KS@GNPs

**Figure 6:**
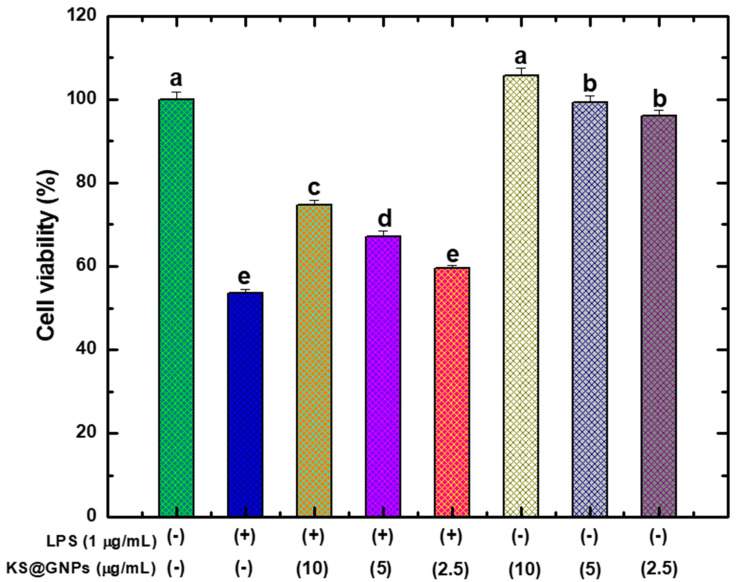
Biocompatibility assessment of KS@GNPs on RAW 264.7 cells. Different concentrations of KS@GNPs (2.5 10 µg/mL) were used for cell treatment for 24 h, with or without LPS (1 µg/mL). WST 1 assay was used to determine cell viability. LPS: lipopolysaccharide. Mean ± SD from triplicate separated experiments is shown. Values expressed by different letters in each column are significantly different in the *t* test (*p* < 0.05).

Biocompatibility of the synthesized KS@GNPs were determined against RAW 264.7 cells. Figure 6 revealed that the cells without any treatment either LPS or KS@GNPs were grown 100%. However, cells were damaged and cell viability was reduced by 46.6% upon treatment with LPS (1 µg/mL). Generally, LPS induces inflammation to the cells which causes cell rupture, cell clumps, and necrosis that were understood by the cell viability reduction. Interestingly, addition of various concentration of KS@GNPs in the LPS induced cells manifested well recovery from cell damage, and the cell variability was increased by 24.4% which indicated that KS@GNPs have no negative effect like LPS. The highest concentration (10 µg/mL) of KS@GNPs stimulated to cell growth which were comparable with the control cells. In addition, over confluence of cell growth were also observed when different concentrations (10, 5, and 2.5 µg/mL) of KS@GNPs alone was treated with RAW 264.7 cells which proved that cell growth recovery from LPS induced cell-damage was absolute.

### 2.8. Anti-Cancer Activity

#### 2.8.1. Cytotoxicity and Cell Viability Assay

**Figure 7:**
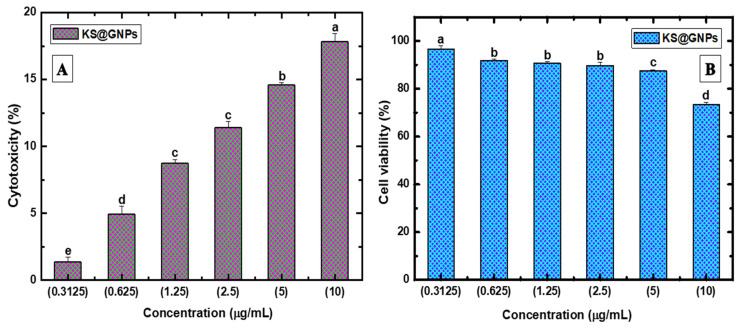

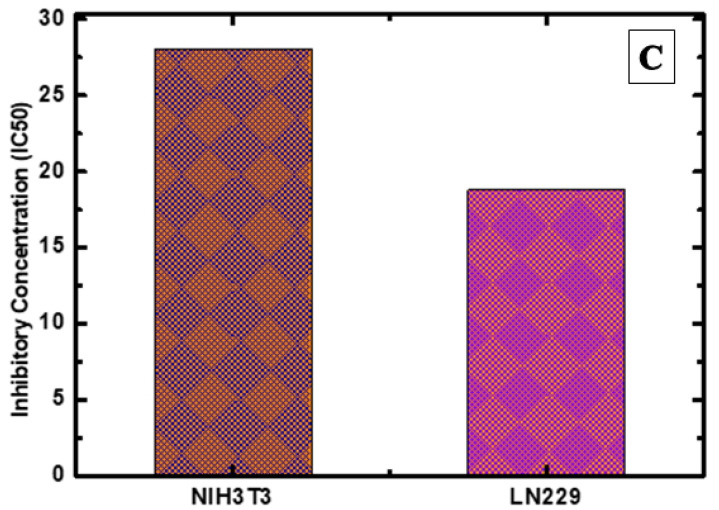
Cytotoxicity of KS@GNPs in normal mouse fibroblast NIH3T3 cells (**A**), cell viability of KS@GNPs in human in human glioma (LN-229) cell line (**B**). Inhibitory concentration (IC_50_) values of normal (NIH3T3) cells and cancer (LN-229) cells (**C**). Mean ± SD from triplicate separated experiments is shown. Values expressed by different letters in each column are significantly different in the *t* test (*p* < 0.05).

Amidst various biosynthesized metal nanoparticles, GNPs might effectively act as an alternative cancer healing agent while its congruity to the non-cancerous cells necessitates being ensured. Hence, we firstly screened the toxic impact of KS@GNPs on the normal cells (NIH3T3: mouse fibroblast cell line) which was examined under different concentrations (0.3125–10 µg/mL) for 24 h by WST assay. Results revealed that KS@GNPs induced cytotoxicity to the healthy cells was dose-dependent fashion but not worthy of remark (Figure 7A). Although just under a fifth (17.82%) of cell growth inhibition was recorded during high concentration treatment when compared to control. In contrast, treatment of similar concentrations of KS@GNPs produced overt cell toxicity in human glioma cells (LN-229) and cell viability was reduced obviously (Figure 7B). The optimum concentration (10 µg/mL) resulted higher cell death and viable cell was notably declined by 26.6%. Furthermore, cell growth inhibitory concentration (IC_50_) of KS@GNPs for NIH3T3 and LN-229 was found 28.05 µg/mL and 18.79 µg/mL, respectively (Figure 7C), and these findings were in line with many previous studies [32,33]. Herein, very low level of IC_50_ values of KS@GNPs were documented, predominantly, KS@GNPs have the sturdiest inhibitory activity against LN-229 cells at very low concentration compared to NIH3T3 cells which might be attributed to either surface plasmon resonance or exclusive physiochemical properties (size and charge) of the synthesized KS@GNPs [34].

#### 2.8.2. KS@GNPs Induced Cellular Changes, Oxidative Stress, and Apoptosis

**Figure 8:**
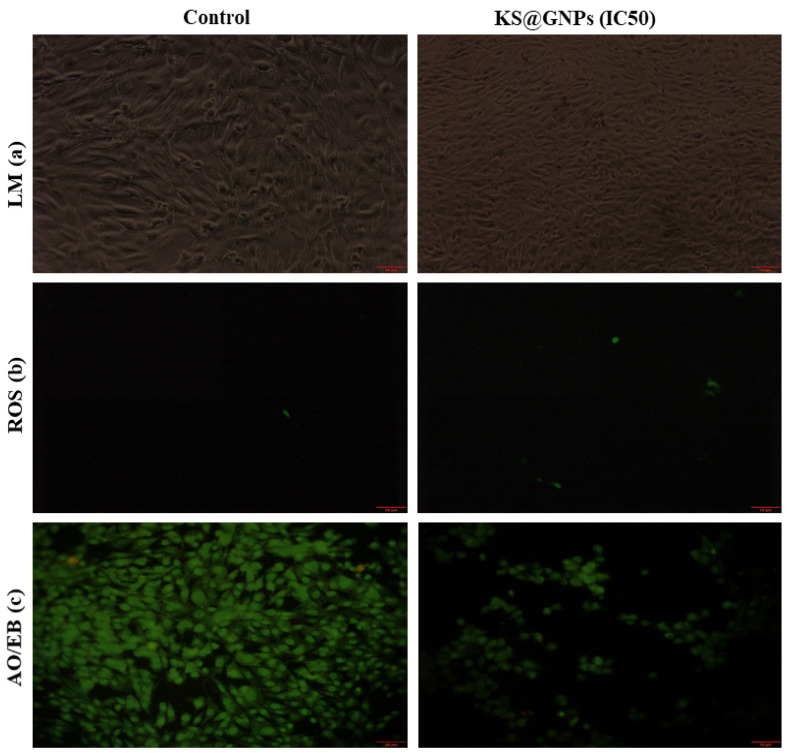
(**a**) Cytotoxicity effect of KS@GNPs on mouse embryo fibroblast cells (NIH3T3) analyzed by WST assay. Morphological changes in NIH3T3 cells, (**a**) light microscopic observation, (**b**) fluorescent microscopic observation to detect ROS generation, and (**c**) AO/EB straining of NIH3T3 untreated (control) and treated with KS@GNPs (Scale bar 50 μm).

**Figure 9:**
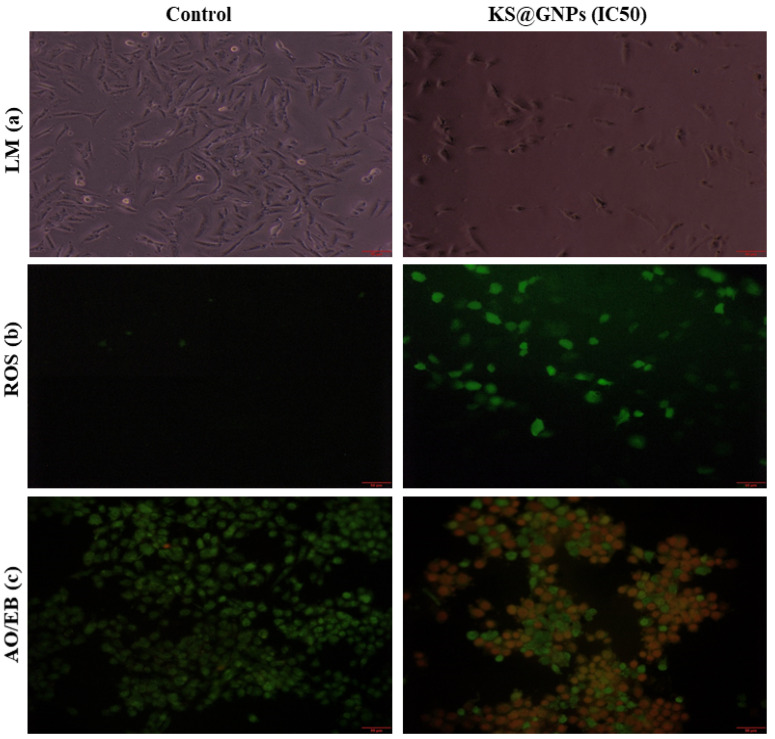
(**a**) Cell viability of KS@GNPs on human glioma cancer cells (LN-229) analyzed by WST assay. Morphological changes in LN-229 cells, (**a**) light microscopic observation, (**b**) fluorescent microscopic observation to detect ROS generation, and (**c**) AO/EB straining of LN-229 untreated (control) and treated with KS@GNPs (Scale bar 50 μm).

The IC_50_ of KS@GNPs induced cellular morphology were determined through the observation under the light and fluorescence microscope. NIH3T3 cells treated with IC_50_ of KS@GNPs showed no obvious morphological discrepancies and cytotoxic structures (Figure 8a) whereas higher cellular damages followed by cell membrane blebbing, cell clump and shrinkage, fragmented and condensation of nuclei, and cell burst were appeared in LN-229 cells (light microscope observation) (Figure 9a). Such morphological divergences are directly interlinked with the oxidative stress mediated reactive oxygen species (ROS) generation within the cells [35].

In order to reassess whether KS@GNPs triggered oxidative stress, the dichloro-dihydro-fluorescein diacetate (DCFH-DA) staining assay was conducted. Figure 8b displayed that IC_50_ of KS@GNPs did not stimulate to ROS generation in NIH3T3 cells thereby no oxidative stress which might be the reason of intracellular antioxidant activity. In contrast, excessive ROS generation with the cells treated with IC_50_ of KS@GNPs was observed which evidenced that inordinate number of ROS production caused by KS@GNPs is the intrinsic pathway of cancer cell death (Figure 9b) [36]. Several key factors of metal NPs including internalization, particle size, charge, and surface area influence higher ROS generation which initiates a sequence of pathological events followed by cell death [37]. Mainly, upon interaction of GNPs with cellular components provoke to release Au^3+^ ions that are accumulated around the cellular organelles (mitochondria, endoplasmic reticulum and DNA) and cause irreparable DNA damage or disruption of the mitochondrial electron-transfer chain [38,39]. Such imbalances result overproduction of free radicles that leads to dropping of glutathione (GSH) into its oxidized form, glutathione disulfide, which initiates oxidative stress and subsequently leads to apoptosis [40].

**Figure 10:**
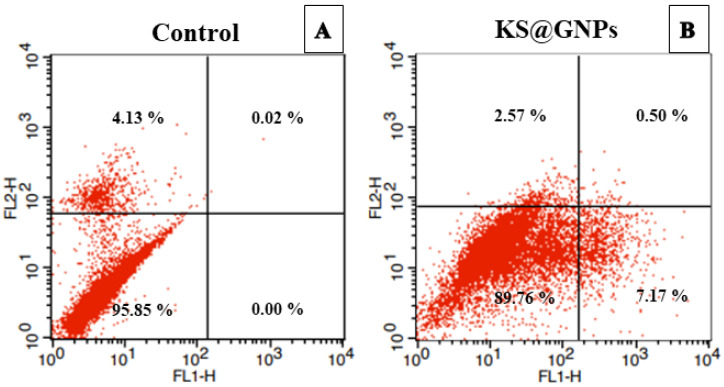
Flow cytometry-based analysis of cell death in human glioma cancer cells (LN-229) untreated (**A**) and treated with KS@GNPs (**B**).

The dual staining (AO/EB) was applied in order to confirm KS@GNPs induced cell death stages (early apoptosis, apoptosis, late apoptosis and necrosis) in NIH3T3 and LN-229 cell lines. AO/EB staining demonstrated light green stain (no cell membrane damage) in both untreated control cells and cells treated with IC_50_ of KS@GNPs of NIH3T3 (Figure 8c). A similar appearance (a typical architecture) was seen in the case of untreated control cells of LN-229 which indicated that the cells remained intact and uninfluenced. However, KS@GNPs treated LN-229 cells exposed an elevated distribution of red color (apoptotic) cells. It can be concluded that in KS@GNPs manifested profound cytotoxic activity against cancerous cell compared to the healthy cell lines (Figure 9c). These outcomes were also verified by FITC/PI mediated flow cytometer-based investigation of cell death stages prompted by the in KS@GNPs action. It was found that untreated LN-229 cells revealed 95.85% live cells while IC_50_ of KS@GNPs treatment 89.76% live cells, 7.17% early apoptosis, 0.50% apoptosis and 2.57% necrosis (Figure 10). These results support KS@GNPs treated cell viability and cell morphological data of this study and are in accordance with our previous study [24].

## 3. Materials and Methods

### 3.1. Chemical and Reagents

Chloroauric acid (HAuCl_4_; 99.99%, Sigma, St. Louis, MO, USA), Lipopolysaccharides (LPS; Isotype 055: B5), water soluble tetrazolium (WST) assay kit (EZ-Cytox, Daeil Lab Service, Seoul, Korea), Phosphate buffered saline (PBS), Dulbecco’s Modified Eagle’s Medium (DMEM), Fetal Bovine Serum (FBS) and penicillin–Streptomycin (PS) from Gibco (Waltham, MA, USA). The human glioma cell-line (LN-229), Swiss albino mouse embryo tissue cell-line (NIH3T3), and Ralph and William’s cell line (RAW 264.7) was collected from the Korean Cell bank Line Bank (KCLB, Seoul, Korea).

### 3.2. Crude Extract Preparation from Kenaf Seed (KS)

Kenaf (*Hibiscus cannabinus*) Seed (collected before harvesting) was provided by the Research Institute of Kenaf Co. Ltd. (Gangwondaehak-gil, Chuncheon-si, Gangwon-do, Korea). The seed was oven-dried with a suitable temperature (50 °C for seven days) in order to reduce moisture content. Later, the dried seed were powdered using a pin crusher (JIC-P10-2; Myungsung Machine, Seoul, Korea). The powders were passed through sieves (mesh size 300 µm) and stored at room temperature. 

For the preparation of the crude extract, 80 g of the kenaf seed (KS) fine powder was soaked in 800 mL of 70% (*v*/*v*) methanol. The solvent extract was placed on a rotary shaker (JEIOTECH SI-900 R) with continuous stirring for 10 days at room temperature. Afterwards, the extracted solvent was filtered (Whatman filter paper No. 1) and evaporated (40 °C) using rotary evaporator in order to get yield (2.4 g; semisolid) of KS extract. The semisolid was powdered by freeze drying at −50 °C and then used to prepare for the stock solution.

### 3.3. Kenaf Seed-Mediated Gold Nanoparticles Synthesis (KS@GNPs)

The prepared KS extract was used to synthesize gold nanoparticles (KS@GNPs) by reducing and capping of HAuCl_4_ under microwave irradiation (MWI). To state the process, KS (1 g) was added to Milli-Q water (100 mL) and sonicated (1 h) and stirred (4 h) to get well-mixed stock solution (1%). Afterwards, stock solution (30 mL) was mixed with HAuCl_4_ (10 mL) in a glass vial (20 mL) and subjected to MW (Midea, MC-E230KW, and 800 W) until the reaction mixture turned into a blushing red color. The final concentration of KS and HAuCl_4_ were 1% and 1 mM, respectively, and MWI time was 90 s. However, the overall synthesis conditions were optimized systemically with the concentration of KS (0.1 to 1%), HAuCl_4_ (0.1 to 1 mM), and MWI time (30 to 90 s) by changing one parameter while keeping other parameters constant. The final biosynthesized KS@GNPs were centrifuged (10 min at 14000 rpm) and the obtained pellets (3 times washed) were freeze dried and preserved for further characterizations.

### 3.4. Characterizations of KS@GNPs

The reaction conditions of KS@GNPs were investigated by UV-visible spectrophotometer (UV-1800 240 V, Shimadzu Corporation, Koyoto, Japan) with 300 to 700 nm wavelength scanning range. The formation of KS@GNPs was confirmed by the Fourier transform infrared spectrophotometer (FTIR) (Perkin-Elmer Model 1600; Norwalk, CT, USA). Here, a pure KBr pellets (2 mg of GNPs were mixed with KBr) was used with the scanning range from 400 to 4000 cm^−1^. The crystallinity of KS@GNPs was analyzed by X-ray diffractometer (X’pert PRO MPD, PANalytical BV, Almelo, The Netherlands) using an operating voltage 45 kV; a current of 40 mA, Cu radiation (1.54430 Å), and at a scanning rate 0.388/min within the region of 2 thetas between 5 and 90 °. The average particle size distribution and the surface charge were determined through a dynamic light scattering (DLS) equipment (Zeta plus 90, Brookhaven Instrument Co., Holtsville, NY, USA). The size, morphology, Energy Dispersive X-ray (EDX) spectrum, and selected electron diffraction (SAED) of KS@GNPs were evaluated by using HR-TEM (LEO-912AB OMEGA, LEO, Freising, Germany). A thin coat of the sample was prepared for TEM analysis by diffusing a drop of KS@GNPs solution on the cupper grid (operating voltage 200 eV). 

### 3.5. Biocompatibility Assessment of KS@GNPs

The biocompatibility study of KS@GNPs was assessed using the water-soluble tetrazolium (WST) assay kit against RAW 264.7 cells (a mouse macrophage cell line). Initially, the cell was cultured (24 h at 37 °C in an incubator with 5% CO_2_) under DMEM medium and then seeded into 96-well plates, and incubated, followed by the above-mentioned conditions. After incubation, DMEM medium was changed and the cells were treated with or without LPS. After 4 h incubation, different concentrations (1.25, 2.5, and 5 μg/mL) of KS@GNPs were induced and incubated again for 24 h. Afterwards, WST reagent was treated to each well, and after incubation (2 h), the absorbance was recorded (450 nm) using a microtiter plate reader [41]. 

### 3.6. Cytotoxicity and Anti-Cancer Activity of KS@GNPs

Using the water-soluble tetrazolium (WST) assay kit, the cytotoxicity (NIH3T3 cells) and anti-cancer (LN-229 cells) activity of KS@GNPs were analyzed. In short, both NIH3T3 and LN-229 cells were cultured under DMEM medium (incorporated with PS in humidified 5% CO_2_ incubator at 37 °C for 24 h). After the incubation, NIH3T3 (5 × 10^4^ cells), and LN-229 (1 × 10^5^ cells) were separately seeded in the 96-well plates and incubated. Later, based on the cells’ confluence (80–90%), KS@GNPs (0.3125, 0.625, 1.25, 2.5, 5, and 10 μg/mL) were treated to the seeded cells and placed for incubation again. Finally, EZ-CyTox reagent (10 μL) was induced to each well and the absorbance was measured at 450 nm. The cytotoxicity of NIH3T3 and cell viability of LN-229 were determined from the absorbance (OD) using the established formula as described elsewhere [24]. The untreated cells (without KS@GNPs treatment) were regarded as control and denoted as CK.

#### 3.6.1. Intracellular Reactive Oxygen Species Determination Induced by KS@GNPs

Through the stain DCFH-DA, the presence of reactive oxygen species (ROS) was determined [24]. Briefly, NIH3T3 and LN-229 cells were treated with KS@GNPs (1000 μg/mL) and incubated for 24 h. Afterwards, the cells were washed with PBS and added 10 μL of DCFH-DA, and again incubated (at 37 °C for 30 min). After washing the incubated cells, the fluorescent intensity was measured by fluorescent spectrometer (Thermo Scientific, Waltham, MA, USA). Additionally, fluorescent microscopy (Olympus CKX53, Tokyo, Japan) was used to observe the cells with excitation wavelength (488 nm) and emission wavelength (525 nm). The cells without KS@GNPs treatment were regarded as control (CK).

#### 3.6.2. Acridine Range (AO)/Ethidium Bromide (EB) Staining

The apoptosis (cell death) of the NIH3T3 and LN-229 cells caused by KS@GNPs was identified through staining method using acridine orange/ethidium bromide (AO/EB) [24]. Briefly, KS@GNPs (1000 μg/mL) were treated with NIH3T3 and LN-229 cells and incubated at 37 °C and 5% CO_2_ for 24 h. Afterwards, both cells were washed (cold PBS) and stained with AO and EB solution and then observed under a fluorescence microscope with a magnification of 20× (Olympus CKX53, Tokyo, Japan). The cells with no treatment (KS@GNPs) were regarded as control (CK).

#### 3.6.3. Evaluation of Apoptosis by Annexin V-FITC/PI Staining

KS@GNPs induced apoptosis in LN-229 cells was evaluated using Annexin V-FITC apoptosis detection kit. Primarily, the seeded cells (2 × 10^5^ cells into 6 well plates) were treated with KS@GNPs (1000 μg/mL) and without treatment of KS@GNPs (control) and then incubated at 37 °C and 5% CO_2_ for 48 h. Afterwards, with the sterile cell scraper, cells were collected and strained with the Annexin V-FITC conjugate and propidium iodide (PI) (incubated in dark for 10 min). Lastly, the apoptotic rates of stained cells were determined by using flow cytometer (Becton-Dickinson, Franklin Lakes, New Jersey, NJ, USA) [24]. 

### 3.7. Statistical Analysis

All data were expressed as mean ± standard deviation (SD) of triplicate measurements. The results were compared using a paired *t*-test to evaluate any substantial differences among concentrations at the 5% level. MINITAB (version 17.0, Minitab Inc., State College, PA, USA) was used to evaluate the paired t-test.

## 4. Conclusions

In summary, we have evidenced the effectiveness of in-situ kenaf-seed mediated microwave-assisted uniform gold nanoparticle (GNPs) synthesis. The overall synthesis processes are accurate, inexpensive, and reliable from both technical and recyclable analytical platforms. Here, functional groups of metabolites such as hydroxyl groups from kenaf seed played a pivotal role of both reducing and supporting agent that phenomenon made the kenaf seed as a sustainable material. The obtained KS@GNPs manifested superior characteristics in terms of high-quality crystal, spherical in shape, amorphous, enhanced colloidal stability, and no agglomeration. KS@GNPs exposure to RAW 264.7 and NIH3T3 cell lines showed utmost relevance and no pathological abnormalities which pinpointed their biocompatibility and biosafety. Further anticancer research confirmed the immense therapeutic value, as KS@GNPs triggered cancer cell (LN-229) death through the induction of higher ROS generation and apoptosis mechanism. However, the exact anticancer mechanisms of the latter remain poorly understood. Hence, additional worthwhile study and particularly animal model experiments are deserved to expound the observed anticancer effect.

## Data Availability

All data and materials used are available in the manuscript.
